# Supplementary material for: CDX2- and PAX8-Expressing Subtypes in Female Urethral Adenocarcinoma: Pathogenesis Insights through Immunohistochemical and Morphological Analyses
Source: Diagnostics (Basel). 2023 Jul 19;13(14):2408. doi: 10.3390/diagnostics13142408 (PMC10377779; doi:10.3390/diagnostics13142408)
Supplement: Supplementary file 1 [file diagnostics-13-02408-s001.zip › diagnostics-2427557-supplementary.pdf]

Table S1. All results of immunohistochemical analyses.

	**Case 1**	**Case 2**	**Case 3**	**Case 4**	**Case 5**	**Case 6**	**Case 7**	**Case 8**	**Case 9**	**Total**	**CDX2-expressing subtype**	**PAX8-expressing subtype**
CD10	+	-	+	±	+	+	-	-	-	5/9(55%)	4/7(57%)	1/2(50%)
GATA3	±	-	-	-	±	-	-	-	-	2/9(22%)	2/7(28%)	0/2(0%)
p16	+	+	+	+	+	+	+	±	+	9/9(100%)	7/7(100%)	2/2(100%)
PSAP	-	-	-	-	±	-	-	-	-	1/9(11%)	1/7(14%)	0/2(0%)
Glypican 3	-	-	±	-	-	-	-	±	±	3/9(33%)	2/7(28%)	1/2(50%)
Uroplakin 2	-	-	-	-	-	-	-	-	±	1/9(11%)	0/7(100%)	1/2(50%)
WT1	-	-	-	-	-	-	-	-	-	0/9(0%)	0/7(100%)	0/2(0%)
TTF1	-	-	-	-	-	-	-	-	-	0/9(0%)	0/7(100%)	0/2(0%)
ER	-	-	-	-	-	-	-	-	-	0/9(0%)	0/7(100%)	0/2(0%)
PgR	-	-	±	-	±	±	±	-	-	4/9(44%)	3/7(42%)	1/2(50%)
HER2	-	-	-	-	-	±	±	±	±	4/9(44%)	2/7(28%)	2/2(100%)
EGFR	±	±	±	±	±	+	+	±	±	9/9(100%)	7/7(100%)	2/2(100%)
PD-L1	-	±	-	-	-	±	-	-	-	2/9(22%)	2/7(28%)	1/2(50%)
PSA	-	-	-	-	-	-	-	-	-	0/9(0%)	0/7(0%)	0/2(0%)
NKX3.1	-	-	-	-	±	-	-	-	-	1/9(11%)	1/7(14%)	0/2(0%)
PAX2	-	-	-	-	-	-	-	-	-	0/9(0%)	0/7(0%)	0/2(0%)
